# Draft Genome Sequences of *Salinivibrio proteolyticus*, *Salinivibrio sharmensis*, *Salinivibrio siamensis*, *Salinivibrio costicola* subsp. *alcaliphilus*, *Salinivibrio costicola* subsp. *vallismortis*, and 29 New Isolates Belonging to the Genus *Salinivibrio*

**DOI:** 10.1128/genomeA.00244-17

**Published:** 2017-07-06

**Authors:** Clara López-Hermoso, Rafael R. de la Haba, Cristina Sánchez-Porro, Sion C. Bayliss, Edward J. Feil, Antonio Ventosa

**Affiliations:** a Department of Microbiology and Parasitology, Faculty of Pharmacy, University of Sevilla, Sevilla, Spain; b Department of Biology and Biochemistry, University of Bath, Bath, United Kingdom

## Abstract

The draft genome sequences of 5 type strains of species of the halophilic genus *Salinivibrio* and 29 new isolates from different hypersaline habitats belonging to the genus *Salinivibrio* have been determined. The genomes have 3,123,148 to 3,641,359 bp, a G+C content of 49.2 to 50.9%, and 2,898 to 3,404 open reading frames (ORFs).

## GENOME ANNOUNCEMENT

The genus *Salinivibrio*, within the class *Gammaproteobacteria*, family *Vibrionaceae*, currently includes four species, one of them with three subspecies. Members of this genus are moderately halophilic bacteria, growing in the presence of 0 to 25% NaCl, and inhabit salted meats, brines, and hypersaline environments (saline lakes and salterns) (1–6). The members of the genus *Salinivibrio* grow optimally at ca. 7.5% NaCl, 37°C (range, 17 to 55°C), and pH 7.5 (range, pH 5 to 11). Therefore, they have developed cellular mechanisms to thrive under extreme conditions, such as high salt concentrations, alkaline pH values, UV radiation, or the presence of arsenic and other metals (3, 7, 8). *Salinivibrio costicola* subsp. *costicola* is considered a representative model for studies on moderately halophilic bacteria, and it has been used for osmoregulation and other physiological studies (9, 10). The genome of this bacterium, as well as those of *Salinivibrio* spp. S10B, S34, and S35, have already been published (8, 11); here, we report the draft genome sequences of the other species or subspecies of this genus, as well as of 29 new strains isolated from pond water of different salterns located in Spain and Puerto Rico (Table 1).

**TABLE 1:** Features of the 34 draft genomes of *Salinivibrio* strains sequenced

Strain	Place of isolation	Genome size (bp)	*N*_50 _(bp)	No. of contigs	Sequencing depth (×)	% G+C	No. of ORFs	Sequencing technology	DDBJ/ENA/GenBank accession no.
*S. costicola* subsp. *alcaliphilus* DSM 19052	Campania, Italy	3,326,316	74,560	104	74	49.3	3,114	Illumina HiSeq	MUFR00000000
*S. costicola* subsp. *vallismortis* DSM 8285	Death Valley, USA	3,478,614	196,230	44	83	49.7	3,218	Illumina HiSeq	MUFQ00000000
*S. proteolyticus* DSM 19052	Bakhtegan Lake, Iran	3,603,496	143,067	51	11	49.8	3,338	Illumina MiSeq	MUFP00000000
*S. sharmensis* DSM 18182	Ras Mohammed Park, Egypt	3,326,895	229,272	40	35	50.4	3,028	Illumina MiSeq	MUFC00000000
*S. siamensis* JCM 14472	Nakornnayok, Thailand	3,442,185	114,651	61	17	50.3	3,126	Illumina MiSeq	MUFB00000000
*Salinivibrio* sp. AL184	Santa Pola, Alicante, Spain	3,398,626	198,024	42	100	50.3	3,132	Illumina HiSeq	MUEK00000000
*Salinivibrio* sp. AR640	Aragonesas, Huelva, Spain	3,256,021	87,861	60	31	49.3	2,962	Illumina MiSeq	MUFD00000000
*Salinivibrio* sp. AR647	Aragonesas, Huelva, Spain	3,281,043	311,807	22	22	49.3	3,002	Illumina MiSeq	MUFE00000000
*Salinivibrio* sp. IB282	Isla Bacuta, Huelva, Spain	3,226,186	40,759	157	11	50.7	3,021	Illumina MiSeq	MUET00000000
*Salinivibrio* sp. IB560	Isla Bacuta, Huelva, Spain	3,492,326	111,814	65	28	50.5	3,194	Illumina MiSeq	MUEM00000000
*Salinivibrio* sp. IB563	Isla Bacuta, Huelva, Spain	3,483,818	42,363	173	16	50.5	3,276	Illumina MiSeq	MUEN00000000
*Salinivibrio* sp. IB574	Isla Bacuta, Huelva, Spain	3,612,537	106,472	69	9	49.8	3,385	Illumina MiSeq	MUFN00000000
*Salinivibrio* sp. IB643	Isla Bacuta, Huelva, Spain	3,123,148	79,381	91	20	49.4	2,898	Illumina MiSeq	MUFF00000000
*Salinivibrio* sp. IB868	Isla Bacuta, Huelva, Spain	3,440,756	206,501	30	29	50.5	3,112	Illumina MiSeq	MUEW00000000
*Salinivibrio* sp. IB870	Isla Bacuta, Huelva, Spain	3,437,860	138,190	42	26	50.5	3,104	Illumina MiSeq	MUEX00000000
*Salinivibrio* sp. IB872	Isla Bacuta, Huelva, Spain	3,641,359	82,702	102	11	49.8	3,398	Illumina MiSeq	MUFO00000000
*Salinivibrio* sp. IC202	Isla Cristina, Huelva, Spain	3,608,911	72,723	111	25	50.5	3,404	Illumina MiSeq	MUEO00000000
*Salinivibrio* sp. IC317	Isla Cristina, Huelva, Spain	3,278,261	21,208	236	8	50.9	3,116	Illumina MiSeq	MUEP00000000
*Salinivibrio* sp. MA351	La Malahá, Granada, Spain	3,308,854	95,545	59	25	49.3	3,000	Illumina MiSeq	MUFG00000000
*Salinivibrio* sp. MA421	La Malahá, Granada, Spain	3,535,899	94,274	70	26	50.4	3,251	Illumina MiSeq	MUER00000000
*Salinivibrio* sp. MA427	La Malahá, Granada, Spain	3,263,514	9,975	513	7	49.3	3,296	Illumina MiSeq	MUFH00000000
*Salinivibrio* sp. MA440	La Malahá, Granada, Spain	3,421,552	59,971	102	35	49.5	3,107	Illumina MiSeq	MUFI00000000
*Salinivibrio* sp. MA607	La Malahá, Granada, Spain	3,352,168	87,592	68	26	49.2	3,043	Illumina MiSeq	MUFJ00000000
*Salinivibrio* sp. ML198	Es Trenc, Mallorca, Spain	3,433,707	163,752	44	24	50.4	3,134	Illumina HiSeq	MUEZ00000000
*Salinivibrio* sp. ML277	Es Trenc, Mallorca, Spain	3,446,273	117,752	73	31	50.4	3,146	Illumina MiSeq	MUEL00000000
*Salinivibrio* sp. ML290	Es Trenc, Mallorca, Spain	3,523,661	168,056	36	15	50.2	3,224	Illumina MiSeq	MUEY00000000
*Salinivibrio* sp. ML318	Es Trenc, Mallorca, Spain	3,361,480	65,785	96	14	50.7	3,080	Illumina MiSeq	MUEQ00000000
*Salinivibrio* sp. ML323	Es Trenc, Mallorca, Spain	3,233,876	132,870	63	16	50.8	2,960	Illumina MiSeq	MUES00000000
*Salinivibrio* sp. ML328A	Es Trenc, Mallorca, Spain	3,392,891	172,729	38	55	50.5	3,124	Illumina HiSeq	MUEU00000000
*Salinivibrio* sp. ML331	Es Trenc, Mallorca, Spain	3,545,121	128,351	60	86	50.4	3,262	Illumina HiSeq	MUEV00000000
*Salinivibrio* sp. PR5	Cabo Rojo, Puerto Rico	3,456,024	68,288	105	23	49.9	3,141	Illumina MiSeq	MUFK00000000
*Salinivibrio* sp. PR6	Cabo Rojo, Puerto Rico	3,443,918	165,099	49	28	50.4	3,140	Illumina MiSeq	MUFA00000000
*Salinivibrio* sp. PR919	Cabo Rojo, Puerto Rico	3,489,646	33,984	176	14	49.9	3,269	Illumina MiSeq	MUFL00000000
*Salinivibrio* sp. PR932	Cabo Rojo, Puerto Rico	3,497,261	90,599	74	31	49.9	3,171	Illumina MiSeq	MUFM00000000

The draft genome sequences of the 34 strains were determined using a whole-genome shotgun strategy (12) with two Illumina sequencing systems, Illumina MiSeq (2 × 300-bp paired-end reads) (Swansea University, United Kingdom) and Illumina HiSeq (2 × 100-bp paired-end reads) (Macrogen, South Korea). The sequencing depth ranged between 7- and 100-fold coverage of the entire genome, and the resulting genome assemblies possessed *N*_50_ values between 9,975 and 311,807 bp. All reads were assembled in a range of 22 to 513 contigs (≥1,000 bp) using A5-MiSeq (13) and were used to identify open reading frames (ORFs) and provide a functional annotation of predicted proteins, rRNAs, and tRNA genes. Genome annotation was performed using RAST (14).

The draft genomes were estimated to contain between 3,123,148 and 3,641,359 bp, with a G+C content between 49.2 and 50.9% and a range of 2,898 to 3,404 putative ORFs (Table 1).

Genes involved in osmoregulation mechanisms included ectoine and hydroxyectoine synthesis genes, transporters for betaine, and other compatible solutes, like proline and choline. A complete set of genes encoding RecBCD helicase/nuclease and UvrABC endonuclease holoenzymes were found; these genes are involved in the recombinational repair of DNA double-strand breaks. Enzyme-encoding genes involved in anaerobic respiration were found, such as arsenate reductase (*asrR*) and flavodoxin reductase (FIR). These halophilic bacteria are widely isolated from aquatic hypersaline environments and salted food products. Thus, the availability of their genome sequences, combined with other physiological and biochemical data, will advance our understanding of haloadaptation and other stress defense mechanisms of halophilic bacteria to extreme conditions.

### Accession number(s).

The draft genome sequences of the 34 strains of *Salinivibrio* have been deposited in DDBJ/ENA/GenBank with accession numbers listed in Table 1.
